# Prevalence of type-specific HPV infection by age and grade of cervical cytology: data from the ARTISTIC trial

**DOI:** 10.1038/sj.bjc.6604324

**Published:** 2008-04-08

**Authors:** A Sargent, A Bailey, M Almonte, A Turner, C Thomson, J Peto, M Desai, J Mather, S Moss, C Roberts, H C Kitchener

**Affiliations:** 1 Division of Cancer Studies and Imaging, University of Manchester, Hathersage Road, Manchester M13 0JH, UK; 2 Department of Virology, Central Manchester and Manchester Children's Hospitals NHS Trust, Oxford Road, Manchester M13 9WL, UK; 3 Non-Communicable Disease Epidemiology Unit, London School of Hygiene and Tropical Medicine, Keppel Street, London WC1E 7HT, UK; 4 Cancer Research UK Epidemiology and Genetics Unit, Institute of Cancer Research, Sutton, Surrey SM2 5NG, UK; 5 Department of Cytology, Central Manchester and Manchester Children's Hospitals NHS Trust, Oxford Road, Manchester M13 9WL, UK; 6 Cancer Screening Evaluation Unit, Institute of Cancer Research, Sutton, Surrey SM2 5NG, UK; 7 Division of Medicine, University of Manchester, Stopford Building, Oxford Road, Manchester M13 9PT, UK

**Keywords:** cervical screening, HPV typing, HPV vaccination

## Abstract

Human papillomavirus (HPV) infection causes cervical cancer and premalignant dysplasia. Type-specific HPV prevalence data provide a basis for assessing the impact of HPV vaccination programmes on cervical cytology. We report high-risk HPV (HR-HPV) type-specific prevalence data in relation to cervical cytology for 24 510 women (age range: 20–64; mean age 40.2 years) recruited into the ARTISTIC trial, which is being conducted within the routine NHS Cervical Screening Programme in Greater Manchester. The most common HR-HPV types were HPV16, 18, 31, 51 and 52, which accounted for 60% of all HR-HPV types detected. There was a marked decline in the prevalence of HR-HPV infection with age, but the proportion due to each HPV type did not vary greatly with age. Multiple infections were common below the age of 30 years but less so between age 30 and 64 years. Catch-up vaccination of this sexually active cohort would be expected to reduce the number of women with moderate or worse cytology by 45%, but the number with borderline or mild cytology would fall by only 7%, giving an overall reduction of 12% in the number of women with abnormal cytology and 27% in the number with any HR-HPV infection. In the absence of broader cross-protection, the large majority of low-grade and many high-grade abnormalities may still occur in sexually active vaccinated women.

Human papillomavirus (HPV) infection has a central role in the aetiology of cervical cancer (Walboomers *et al*, 1999). More than 100 HPV types have been described (de Villiers *et al*, 2004) and 40 can infect the anogenital tract. Genital HPV types are categorised according to their association with cervical cancer (Munoz *et al*, 2003). About 20 are classified as high-risk (HR) types and are associated with cervical cancer and precancerous lesions, as well as low-grade cervical pathology. Worldwide, HPV types 16 and 18 cause approximately 70% of cervical cancers; HPV types 31, 33, 35, 45, 52 and 58 account for an additional approximately 20% of cases, although there is substantial geographical variation in the relative frequency of different HR types (Clifford *et al*, 2005). Low-risk HPV types, including HPV6 and 11, cause low-grade cervical lesions, genital warts and recurrent respiratory papillomatosis.

In 2006, a quadrivalent prophylactic vaccine against HPV types 6, 11, 16 and 18 was licensed in the United States (FDA, 2006) and Europe (EMEA, 2006). More recently, the bivalent vaccine against HPV types 16 and 18 has been approved in Europe (EMEA, 2007). Prophylactic HPV vaccination of young female adolescents has been approved as a public health policy in the United Kingdom and other countries, and data on the type-specific prevalence of HPV infection are relevant in assessing its potential impact on cervical pathology and screening.

A Randomised Trial in Screening to Improve Cytology (ARTISTIC) is being conducted within the routine NHS Cervical Screening Programme in Greater Manchester to evaluate the effectiveness of HPV testing in primary cervical screening. The randomised comparison will determine whether combining HPV testing with liquid-based cytology offers added sensitivity over cytology alone and, if so, whether this would be cost-effective. Our first report described the baseline prevalence of HPV16, 18 and all other HR-HPV types in relation to age, cytology and histology (Kitchener *et al*, 2006). We now report type-specific prevalence data for all HR-HPV types in relation to cervical cytology and age.

## MATERIALS AND METHODS

### High-risk HPV detection

After processing for cytology, residual ThinPrep® liquid-based cytology samples were sent to the Virology Laboratory at Manchester Royal Infirmary. High-risk HPV detection was carried out using the Digene Hybrid Capture 2® (hc2) test according to the manufacturer's instructions, as described previously (Kitchener *et al*, 2006). A positive hc2 result was defined as RLU/Co⩾1, according to the manufacturer's criteria at the start of the study. Residual cells from all liquid-based cytology samples were pelleted and stored at −70°C.

### HPV polymerase chain reaction and genotyping

Genotyping of all hc2-positive samples was carried out using the prototype Roche Line Blot Assay (LBA). Polymerase chain reaction (PCR) amplification and product detection for 37 anogenital types were essentially performed as described previously (Peyton *et al*, 2001). Following DNA extraction from a 50 *μ*l volume of the stored, pelleted sample using the Roche MagNA Pure automated system, a 50 *μ*l volume of extracted DNA was added to an equal volume of reaction mixture containing 10 mM Tris-HCl (pH 8.5), 50 mM KCl, 4 mM MgCl_2_, 500 nM each of biotinylated HPV primers PGMY09 and PGMY11, 25 nM each of biotinylated human beta-globin primers PC04 and GH20, 200 *μ*M each of nucleotides dATP, dCTP and dGTP, 600 *μ*M of nucleotide dUTP and 7.5 U of Amplitaq Gold. Following heating at 95°C to activate the Amplitaq Gold polymerase enzyme and render the native DNA single stranded, nucleic acid was amplified by 40 cycles of 95°C/30 s to allow strand denaturation, 55°C/1 min for primer annealing and 72°C/1 min for primer extension. This cycling program was followed by an elongated primer extension period of 56 min at 72°C. Amplified product was stored at 4°C. Following denaturation, amplified product was hybridised to oligonucleotide-coated genotyping strips before colour development and interpretation using the template provided.

Due to the unexpectedly large number of hc2-positive LBA-negative samples, a subset (*n*=102) of these samples was examined using GP5+/6+ PCR (de Roda Husman *et al*, 1995). The PCR product was visualised after electrophoresis through a 2% agarose gel and ethidium bromide staining.

### Cytology

All cytology was read independently of the HPV results. Slides were read according to the routine laboratory protocol and reported as such. There was no attempt to reach consensus for any of the smear grades. There has been no *post hoc* review of cytology. National guidelines were adhered to, which meant that high-grade abnormalities, that is, moderate and severe dyskaryosis, were referred for colposcopy and biopsy. In women with low-grade abnormalities, that is, borderline and mild dyskaryosis, cytology was repeated at 6 months with referral to colposcopy if the abnormality persisted.

## RESULTS

A total of 24 510 eligible women had satisfactory cytology and HPV results by hc2 at entry. Samples from 3813 women (15.6% of all eligible women) were HPV-positive by hc2, but 40 (1.0%) of these either gave negative results for beta-globin gene amplification and were reported as inhibitory or were of insufficient volume for further testing. These 40 were excluded, and all the results were based on the remaining 24 470 women. Crossreactivity with low-risk or HR types not included in the hc2 probe mix was observed in 417 (11.1%) hc2-positive samples. A broad range of HPV type crossreactivity occurred. This was particularly noticeable for HPV types 53, 66 and 70, which were frequently detected. A further 772 (20.5%) hc2-positive samples did not hybridise to any of the LBA probes. These 1189 samples were classified as hc2 positive but HR-HPV negative. The remaining 2584 hc2-positive samples (68.5%) were positive by LBA for one or more of the 13 HR types included in the hc2 HR probe mix. Of those hc2-positive samples giving a low RLU/Co value between 1 and 3, 26.7% contained an hc2 HR type; 16.2% crossreacted with other types and 57.1% failed to type. The corresponding figures of those hc2-positive samples giving a high RLU/Co value of ⩾100 were 91.9, 4.8 and 3.3%. In total, 50% of hc2-positive LBA-negative samples had an RLU/Co value between 1 and 2.11. On testing a subset of 102 hc2-positive LBA-negative samples by GP5+/6+ PCR, 39.2% were found to be HPV positive. Multiple HR-HPV types were detected in 680 (18.0% of hc2-positive samples) and infection with a single HR-HPV type was detected in 1904 (50.5%) samples.

Prevalence rates for each HR-HPV type are shown in Table 1, both overall and by age group. The most common genotype at all ages was HPV16 (overall prevalence 3.3%), followed by HPV types 52 (1.5%), 18 and 31 (both 1.3%), 51 (1.2%) and 39 (1.1%). There was a marked decline in the prevalence of HR-HPV with age, both overall (27.3% below age 30 years and 6.1% at age 30 years or above) and for each HPV type, but less so for hc2-positive samples in which no HR-HPV was detected (6.4% of women aged below 30 years and 4.5% aged 30–64 years).

The HPV prevalence rates by age group and cytology are shown in Table 2 for HPV16, HPV18 without HPV16 and for other HR-HPVs combined. Below age 30 years, a high proportion of infected women carried two or more different HR-HPV types (44% of women with HPV16, 50% with HPV18 but not HPV16 and 24% of all women with other HR-HPVs). Multiple infections were less common at age 30–64 years (23% of women with HPV16, 20% with HPV18 and 14% of women with other HR-HPVs). The proportion with moderate dyskaryosis or worse was 15.3% (396 out of 2584) in women with any HR-HPV infection, 1.2% for hc2 positives with no HR-HPVs and 0.22% for hc2-negative women. The risk of moderate or worse cytology was highest in women infected with HPV16 irrespective of the presence of other HPVs (26.2% for HPV16 alone, 25.3% together with other HR-HPVs).

**Figure 1:**
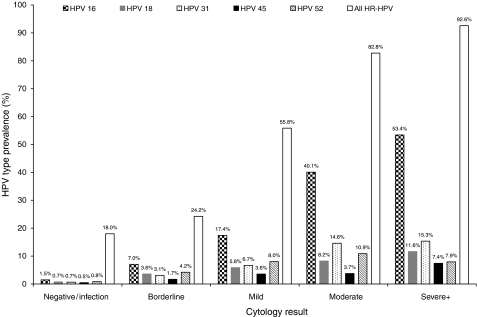
Prevalence rates for four of the commonest five types and HPV45 by cytological grade.

Cytology by HPV status is shown in Table 3 for women aged 20–29 years, 30–64 years and overall. Summing up the number of different HR-HPV types detected in each woman for the denominator, the proportion of all detected infections that were due to each HPV type did not vary greatly with age. Below age 30 years, 24.0% (499 out of 2077) of HR-HPV infections were due to HPV16, compared with 21.3% (306 out of 1435) at age 30–64 years (*P*=0.06). The corresponding proportions were 6.3 and 3.7% for HPV33 (*P*=0.001), 2.4 and 4.1% for HPV35 (*P*=0.003) and 4.5 and 6.7% for HPV45 (*P*=0.005). No other type showed significant variation with age. The proportion of women with a single HR-HPV type who had moderate or worse cytology (Table 3; extreme right column) was 26% for HPV16, between 12 and 19% for HPV types 18, 31, 33 and 58, 7–9% for types 35, 45, 51 and 52, and less than 5% for types 39, 56, 59 and 68. The proportion with borderline or mild cytology was much less variable, ranging from 23 to 42%. The proportion of different grades of cytology positive for HPV types 16, 18, 31, 45 and 52 are shown in Figure 1. This graphically demonstrates the increasing prevalence with cytology grade.

Of the CIN2+ lesions found before the exit round, 108 out of 329 (33%) and 83 out of 225 (37%), respectively, were identified in high- and low-grade cytological abnormalities, which were HR-HPV positive but types 16/18 negative.

## DISCUSSION

The ARTISTIC trial cohort (see Appendix for ARTISTIC Trial Study Group) is the first large population of women in the United Kingdom to have undergone HPV testing and genotyping. Although from a limited geographic area, the setting in primary care makes this a representative population of women across the cervical screening age range. Several conclusions can be inferred from the distributions of each HR-HPV type in relation to age and the cytological findings presented here. The high proportion of women with abnormal cytology who are HR-HPV positive but HPV16/18 negative is clinically significant, as it accounts for 35% (33% of moderate or worse and 37% of borderline or mild) of all of CIN2+ lesions.

The five most prevalent types (16, 18, 31, 51 and 52) together account for 60% of the 3512 HR-HPV infections detected (Table 3); HPV16 and 18 account for 32%. The overall prevalence of HR-HPV infection decreased sharply with age, from 27% below age 30 to 10% at age 30–39 years, 4.2% at 40–49 years and 2.5% at 50–64 years. The prevalence in Manchester between 1988 and 1993 was about 40% lower at each age (16% at age 20–29 years and less than 3% above age 40 years) (Peto *et al*, 2004). The difference in prevalence may be due to lower assay sensitivity or suboptimal specimen collection and storage methods employed in the earlier study. Alternatively or additionally, changes in the prevalence of genital HPV infections as suggested by increased UK diagnosis of genital warts between 1972 and 2005 (Health Protection Report, 2007) may be relevant. However, changes in clinical practices in the diagnosis and reporting of genital warts may further complicate the picture. The difference in prevalence between young and older women is less marked in most other countries (Franceschi *et al*, 2006). Most HR-HPV types show a similar age distribution, with relatively minor differences in the type distribution above and below the age of 30 years. The most marked difference was shown by HPV33, being detected in 9.3% of women with HR-HPV below age 30 years and only 4.5% at older ages (Table 3; *P*<0.001).

The failure of the LBA to confirm that 31.5% of the hc2-positive samples contain hc2 HR-HPV types is a cause of concern, especially if this assay was to be used as a frontline screening test. This is due, in part, to the demonstrated crossreaction of the hc2 test with other putative HR as well as low-risk types. However, the fact that 20.5% failed to yield any detectable HPV type is more problematic. As the whole of the PGMY-amplified product generated during LBA testing was denatured, further analysis by gel electrophoresis was not possible. Further work using another well-documented primer system (GP5+/6+ PCR) followed by gel electrophoresis suggested that 39.2% of these hc2-positive LBA-negative samples might in fact contain HPV. Confirmation of this observation would require further analysis on a larger number of samples. The 13% originally selected merely provides an insight into the true HPV status of these samples. The use of the improved, commercially available linear array assay (Coutlee *et al*, 2006) to confirm these hc2-positive samples should improve the confirmatory rate. There would still remain a substantial number of samples that do not appear to contain a demonstrable HPV genotype. Approximately half of these samples give an hc2 RLU value between 1 and 2 providing further evidence that it may be advisable to raise the hc2 cutoff level, as has been suggested previously (Hesselink *et al*, 2006). A recent study also found that a number of hc2-positive samples do not contain HR types when tested by the LBA, particularly those having low RLU values (Castle *et al*, 2008).

Differences in the relative frequencies of different HPV types are seen both between and within continents. The gross international differences between HPV subtypes (Yamada *et al*, 1997) indicate that infections often involve viruses that have evolved in the region over many centuries, but now there is substantial intercontinental mixing through increased migration. Among HR-HPV-positive women with normal cytology, the relative frequencies for several of the common HPV types were similar to those in other European countries, as reported by Clifford *et al* (2005), although the proportion in our study was substantially greater for HPV52 and for the combined total of types 39, 51, 59 and 68. In a recent study of urine samples from American women aged 18–25 years, the distribution between the 13 HR-HPVs detected by hc2 was also similar to that seen in Manchester, with HPV16 being twice as prevalent as any other type, followed by types 51, 52, 39, 59 and 18 (Manhart *et al*, 2006). A strikingly different distribution was recently reported among 1921 American women aged 14–59 years, with HPV types 52, 59 and 51 being more common than HPV16 (Dunne *et al*, 2007). Although the prototype Roche LBA was used in both studies, the variation in type distribution observed may reflect the different sample types used, self-sampling cervico-vaginal specimens being used in the US study.

The proportion of women with HPV16 who had borderline or mild cytology was increased by the presence of other HR-HPVs (Table 2; 28.3% for single infections and 45.3% for multiple infections), but the proportion with moderate or worse cytology was not (26.2% for single infections and 25.3% for multiple infections). This suggests that HPV16 may compete with less virulent types in the progression to neoplasia rather than in the normal infective process.

The data in Table 2 provide a basis for modelling the overall effect of vaccination on cervical cytology. The simplest assumption is that elimination of HPV16 and 18 would give women with either or both of these viruses, but no other HR-HPVs, the cytological profile of those with no HR-HPVs, with 5% remaining hc2 positive and the remainder becoming hc2 negative, whereas those also infected with other HR-HPVs would move to the category of HR-HPV without HPV16 or 18. Based on this, the number with moderate or worse cytology would be reduced by 45% in a population with this age distribution, but the number with borderline or mild cytology would fall by only 7%, giving an overall reduction of 12% in the number with abnormal cytology and reducing the number with any HR-HPV by 27%. The cost-benefit balance of vaccination would of course be greatly improved if a polyvalent vaccine reduced the risk to a level where it would be safe to carry out routine screening for vaccinated women less frequently.

The impact of vaccination on cytological abnormality rates will be considerably less in women aged over 30 years, as a far lower proportion of low-grade cytological abnormalities are HR-HPV-positive in older women. Human papillomavirus types 16 and/or 18 were detected in 260 out of 930 (28%) women aged below 30 years with low-grade (borderline or mild) cytology, and in only 111 out of 1720 (6.5%) aged 30–64 years. In the absence of broader cross-protection, the large majority of low-grade and many high-grade abnormalities would still occur in vaccinated women. This is consistent with the data emerging from clinical trials of prophylactic vaccine, which show much greater efficacy in preventing CIN2+ than for low-grade abnormalities. Potential cross-protection involving HPV31 and 45 has been demonstrated by Harper *et al*, 2006. Effective cross-protection involving these types could prevent a significant further proportion of both low- and high-grade abnormalities, particularly among women below 30 years, although this needs to be borne out in ongoing clinical trials. So far only limited data are available regarding the effects of cross-protection in relation to clinical outcome (Brown *et al*, 2007). The extent to which vaccines directed against types 16 and 18 would prevent abnormalities associated with non-vaccine types as part of a multiple infection is not yet clear. Only 57% of infections due to HPV16 or 18 in low-grade cytology, and 66% in high-grade cytology, involved no other HR-HPVs.

More detailed age-specific analysis of these data will help to validate models of the possible impact of vaccination on subsequent cervical screening before long-term follow-up of current trial cohorts. The follow-up of ARTISTIC patients to the subsequent routine screening rounds will also provide estimates of type-specific risk over 3–6 years in women with normal baseline cytology.

## Figures and Tables

**Table 1:** Prevalence of HR-HPVs overall and as a proportion of HR-HPV-positive women by age group

	**20–29 years**			**30–39 years**			**40–49 years**			**50–64 years**			**All ages**		
**Type**	***n***	**% of all women**	**% of HR-HPV+ women**	***n***	**% of all women**	**% of HR-HPV+ women**	***n***	**% of all women**	**% of HR-HPV+ women**	***n***	**% of all women**	**% of HR-HPV+ women**	***n***	**% of all women**	**% of HR- HPV+ women**
16	499	9.7	35.5	207	2.7	26.6	62	1.0	23.9	37	0.7	26.1	805	3.3	31.2
18	191	3.7	13.6	89	1.2	11.4	20	0.3	7.7	19	0.3	13.4	319	1.3	12.3
31	185	3.6	13.2	102	1.3	13.1	27	0.4	10.4	12	0.2	8.5	326	1.3	12.6
33	130	2.5	9.3	41	0.5	5.3	10	0.2	3.9	2	0.04	1.4	183	0.7	7.1
35	49	1.0	3.5	39	0.5	5.0	17	0.3	6.6	3	0.1	2.1	108	0.4	4.2
39	159	3.1	11.3	75	1.0	9.6	17	0.3	6.6	15	0.3	10.6	266	1.1	10.3
45	94	1.8	6.7	58	0.8	7.4	20	0.3	7.7	18	0.3	12.7	190	0.8	7.4
51	189	3.7	13.5	73	1.0	9.4	26	0.4	10.0	17	0.3	12.0	305	1.2	11.8
52	211	4.1	15.0	106	1.4	13.6	39	0.6	15.1	11	0.2	7.8	367	1.5	14.2
56	98	1.9	7.0	58	0.8	7.4	13	0.2	5.0	13	0.2	9.2	182	0.7	7.0
58	97	1.9	6.9	46	0.6	5.9	18	0.3	7.0	7	0.1	4.9	168	0.7	6.5
59	125	2.4	8.9	40	0.5	5.1	23	0.4	8.9	11	0.2	7.8	199	0.8	7.7
68	50	1.0	3.6	30	0.4	3.9	8	0.1	3.1	6	0.1	4.2	94	0.4	3.6
16 and/or 18	654	12.7	46.6	289	3.8	37.1	80	1.3	30.9	54	0.96	38.0	1077	4.4	41.7
Any HR-HPV	1404	27.3	100	779	10.3	100	259	4.2	100	142	2.5	100	2584	10.6	100
hc2+ No HR-HPV	329	6.4	—	368	4.8	—	270	4.4	—	222	4.0	—	1189	4.9	—
hc2−	3417	66.4	—	6452	84.9	—	5582	91.3	—	5246	93.5	—	20 697	84.6	—
All women	5150	100	—	7599	100	—	6111	100	—	5610	100	—	24 470	100	—

HR-HPV=high-risk human papillomavirus.

**Table 2:** Prevalence of single and multiple infections with HPV16, 18 and other HR-HPV types by age group and cytology result

		**HPV16**		**HPV18 not 16**		**HPV16 and/or 18**		**hc2 positive**			
	**No. of women**	**Single HR-HPV *n* (%)**	**Multiple HR-HPV *n* (%)**	**Single HR-HPV *n* (%)**	**Multiple HR-HPV *n* (%)**	**No other HR *n* (%)**	**Other HR *n* (%)**	**HR-HPV, not 16/18 *n* (%)**	**HR-HPV *n* (%)**	**No HR HPV *n* (%)**	**hc2 negative *n* (%)**
*Age*											
20–29	5150	280 (5.4)	219 (4.3)	77 (1.5)	78 (1.5)	374 (7.3)	280 (5.4)	750 (14.6)	1404 (27.3)	329 (6.4)	3417 (66.3)
30–39	7599	158 (2.1)	49 (0.6)	65 (0.9)	17 (0.2)	225 (3.0)	64 (0.8)	490 (6.5)	779 (10.3)	368 (4.8)	6452 (84.9)
40–49	6111	51 (0.8)	11 (0.2)	15 (0.2)	3 (0.1)	68 (1.1)	12 (0.2)	179 (2.9)	259 (4.2)	270 (4.4)	5582 (91.3)
50–64	5610	27 (0.5)	10 (0.2)	14 (0.2)	3 (0.05)	43 (0.8)	11 (0.2)	88 (1.6)	142 (2.5)	222 (4.0)	5246 (93.5)
											
*Cytology*											
Negative	21 364	235 (1.1)	85 (0.4)	96 (0.5)	47 (0.2)	340 (1.6)	123 (0.6)	807 (3.8)	1270 (6.0)	940 (4.4)	19 154 (89.7)
B/M	2650	146 (5.5)	131 (4.9)	53 (2.0)	41 (1.6)	210 (7.9)	161 (6.1)	547 (20.6)	918 (34.6)	235 (8.9)	1497 (56.5)
Mod+	456	135 (29.6)	73 (16.0)	22 (4.8)	13 (2.9)	160 (35.1)	83 (18.2)	153 (33.6)	396 (86.8)	14 (3.1)	46 (10.1)
Total	24 470	516 (2.1)	289 (1.2)	171 (0.7)	101 (0.4)	710 (2.9)	367 (1.5)	1507 (6.2)	2584 (10.6)	1189 (4.9)	20 697 (84.6)

B/M=borderline/mild dyskaryosis; HR-HPV=high-risk human papillomavirus; Mod+=moderate dyskaryosis or worse.

**Table 3:** Cytology by HPV status

	**Cytology**												
	**20–29 years**			**30–64 years**			**All ages**				**1904 women with a single HPV infection**		
**HPV type**	**Negative**	B/M*	Mod+*	**Negative**	**B/M**	**Mod+**	**Negative**	**B/M**	**Mod+**	**Total**	**Negative**	**B/M**	**Mod+**
16	184	202	113	136	75	95	320 (39.8%)	277 (34.4%)	208 (25.8%)	805 (100%)	235 (45.5%)	146 (28.3%)	135 (26.2%)
18	93	74	24	67	41	20	160 (50.2%)	115 (36.1%)	44 (13.8%)	319 (100%)	96 (56.1%)	53 (31.0%)	22 (12.9%)
31	83	67	35	62	46	33	145 (44.5%)	113 (34.7%)	68 (20.9%)	326 (100%)	97 (53.3%)	53 (29.1%)	32 (17.6%)
33	45	58	27	21	22	10	66 (36.1%)	80 (43.7%)	37 (20.2%)	183 (100%)	32 (41.6%)	31 (40.3%)	14 (18.2%)
35	23	20	6	28	25	6	51 (47.2%)	45 (41.7%)	12 (11.1%)	108 (100%)	31 (52.5%)	23 (39.0%)	5 (8.5%)
39	67	71	21	56	38	13	123 (46.2%)	109 (41.0%)	34 (12.8%)	266 (100%)	78 (60.5%)	46 (35.7%)	5 (3.9%)
45	47	33	14	58	28	10	105 (55.3%)	61 (32.1%)	24 (12.6%)	190 (100%)	59 (64.8%)	24 (26.4%)	8 (8.8%)
51	70	99	20	63	40	13	133 (43.6%)	139 (45.6%)	33 (10.8%)	305 (100%)	83 (50.6%)	69 (42.1%)	12 (7.3%)
52	96	88	27	82	57	17	178 (48.5%)	145 (39.5%)	44 (12.0%)	367 (100%)	105 (56.1%)	68 (36.4%)	14 (7.5%)
56	43	46	9	40	38	6	83 (45.6%)	84 (46.2%)	15 (8.2%)	182 (100%)	48 (53.3%)	38 (42.2%)	4 (4.4%)
58	40	45	12	32	24	15	72 (42.9%)	69 (41.1%)	27 (16.1%)	168 (100%)	49 (57.6%)	24 (28.2%)	12 (14.1%)
59	69	44	12	46	20	8	115 (57.8%)	64 (32.2%)	20 (10.1%)	199 (100%)	74 (69.8%)	27 (25.5%)	5 (4.7%)
68	27	19	4	27	13	4	54 (57.4%)	32 (34.0%)	8 (8.5%)	94 (100%)	34 (72.3%)	11 (23.4%)	2 (4.3%)
16 and/or 18	265	260	129	198	111	114	463 (39.8%)	371 (34.4%)	243 (22.6%)	1077 (100%)	—	—	—
Any HR-HPV	651	556	197	619	362	199	1270 (43.0%)	918 (35.5%)	396 (15.3%)	2584 (100%)	—	—	—
hc2+ No HR-HPV	236	88	5	704	147	9	940 (49.1%)	235 (19.8%)	14 (1.2%)	1189 (100%)	—	—	—
hc2−	3119	286	12	16 035	1211	34	19 154 (79.1%)	1497 (7.2%)	46 (0.22%)	20 697 (100%)	—	—	—
All women	4006	930	214	17 358	1720	242	21 364 (92.5%)	2650 (10.8%)	456 (1.9%)	24 470 (100%)	1021	613	270
No. of HR-HPVs detected	887	866	324	718	467	250	1605	1333	574	3512	1021	613	270

B/M=borderline/mild dyskaryosis; HR-HPV=high-risk human papillomavirus; Mod+=moderate dyskaryosis or worse.

Results by age (20–29, 30–64 years), overall, and in 1904 women with a single HPV type.

## APPENDIX

*ARTISTIC Trial Study Group:* HC Kitchener (principal investigator), C Thomson (trial coordinator); *Epidemiology/Statistics:* J Peto, M Almonte, C Gilham, C Roberts, S Moss; *Cytopathology:* M Desai, J Mather; *Health Economics:* R Dowie, B Stoykova; *Virology Studies:* A Bailey, A Sargent, A Turner.
